# Are all primary retinal detachments the same? Anatomic and functional differences between phakic and pseudophakic patients

**DOI:** 10.1186/s40942-023-00455-y

**Published:** 2023-03-26

**Authors:** Ane Gibelalde, Sergio Pinar-Sueiro, Oliver Ibarrondo, Itziar Martínez-Soroa, Javier Mendicute, Miguel Ruiz Miguel

**Affiliations:** 1 Department of Ophthalmology, Donostia University Hospital, Paseo del Dr Beguiristain sn. San Sebastian, 20014 Donostia San-Sebastian, Gipuzkoa Spain; 2 AP-OSI Research Unit, Alto Deba Integrated Health Care Organization, Mondragon, Spain

**Keywords:** Pseudophakic retinal detachment, Phakic retinal detachment, Macula, Posterior vitreous detachment

## Abstract

**Background:**

Given differences in the pathogenic mechanisms underlying primary retinal detachment (RD) as a function of the status of the lens, the objective was to explore differences between pseudophakic and phakic patients with primary RD.

**Methods:**

A retrospective study including 821 patients who underwent surgery for RD [491 cases of phakic and 330 of pseudophakic RD (pRD and psRD, respectively)] in our hospital between 2012 and 2020.

**Results:**

The mean age was 58.24 ± 12.76 years in the pRD group and 66.87 ± 11.18 years in the psRD group (p = 0.001). There were more men in both groups (70% and 64.23% of pseudophakic and phakic patients, respectively; p = 0.07). The most common location for the RD was superior in both groups (43.94% and 51.93% of pseudophakic and phakic patients, respectively), rates of inferior and total RD were somewhat higher in the psRD group (31.82% and 13.33% in pseudophakic vs 25.25% and 11.0% in phakic patients, p = 0.001). In pseudophakic and phakic patients respectively, macular involvement in 69.09% and 62.73% of cases (p = 0.067). Proliferative vitreoretinopathy was significantly more common in the psRD group (7.88% vs 3.6% in phakic patients, p = 0.01).The rate of final anatomic reattachment differed markedly between groups, with a higher rate in phakic (94.03%) than pseudophakic (87.27%) patients (p = 0.001).

**Conclusions:**

The specific pathogenic mechanism involved in psRD seems to be responsible for worse evolution characteristics which are associated with poorer final anatomic and functional outcomes in pseudophakic patients.

## Key messages

**
What is known**

Retinal detachment is a complication that leads to blindness without an early adequate treatment.

There is a noticeable increase in the number of phacoemulsification surgeries performed due to a higher life expectancy and the boom of phacorefractive surgery that will lead to a growing incidence of pseudofaquic RD ( psRD). It is important to know the pathophysiology of pseudophakic retinal detachment considering a worse prognosis in psDR compared to phakic RD.

**What is new**

There are not many studies comparing both groups of retinal detachment considering lens status and none with such a high data. Our results found remarkable differences the anatomic and structural evolution with the poorer prognosis in the pseudophakic group. We believe that is neccesary to give a properly inform patients asking for phacorefractive surgery or cataract of the risks according to the age and other epidemiologic factors, considering a worse prognosis in PRD compared to phakic RD.

## Introduction

Retinal detachment (RD) is a medical condition that can potentially lead to blindness, and rhegmatogenous RD is the most common type [1]. The prevalence of this condition in the general population with no history of surgery or eye trauma is around 5.3 and 12.6 per 100,000 (0.08%). [2–4]. Although pseudophakic RD (psRD) is a rare late complication of phacoemulsification, the incidence of RD is as high as 0.7% [0.6–1.7%] in pseudophakic patients, which is markedly higher than that of rhegmatogenous RD observed in the general population. [5–7]. The pathogenic mechanisms involved in these types of RD differ depending on the status of the crystalline lens.

Posterior vitreous detachment (PVD) is a physiological process associated with age, characterized by progressive vitreous liquefaction which rapidly increases between the sixth and seventh decades of life [8]. To trigger physiological PVD, this liquefaction process that occurs over the years is insufficient; there also needs to be a weakening of the vitreous cortex-to-internal limiting membrane adhesion. During the cataract surgery, there are anatomic and biochemical changes in the vitreous body that lead to a condition called anomalous PVD, given that it is not associated with the necessary weakened internal limiting membrane adhesion, creating stronger pulling forces on the retina. This explains why patients under 60 years of age, who lack the protective effect of vitreous detachment, have a significantly higher incidence of psRD, with rates as high as 3.5% [9]. This study focuses on comparing the characteristics of these two types of RD.

## Materials and methods

### Study design

We conducted a retrospective study using data from the electronic health records of patients who underwent RD surgery between 2012 and 2020 in Donostia University Hospital, part of the Basque Public Health Network (Osakidetza). The inclusion criteria were having primary RD and undergoing surgery during the study period. Patients with a history of penetrating eye injury, RD secondary to macular holes and/or a history of eye surgery other than phacoemulsification were excluded.

Two large groups of patients were established as a function of the status of the crystalline lens at the time of the surgery, patients being classified as phakic or pseudophakic. We collected data on the following variables in both groups: age, sex, date of phacoemulsification surgery in pseudophakic patients, intraoperative complications, YAG-laser capsulotomy in the years after phacoemulsification and prior to vitrectomy for RD, date of retinal surgery, surgical technique, macular status at diagnosis, extension of the RD, placement of the retinal tear, based on data extracted from the clinical records (anatomo-functional description made by the ophthalmologist who made the initial diagnostic exploration, as well as findings from the surgical procedure), considering upper retinal tears, those located over the horizontal meridian between the 3 and 9 o´clock positions, and inferior tears, those placed on the opposite side, inferior to the horizontal meridian. There were also collected data regarding recurrence after the first surgery, number of recurrences, and eye axial length (AL) in mm as measured by optical biometry (IOLMaster 500, Zeiss). Age was categorized by decade and AL into three groups: < 23.5 mm, ≥ 23.5 mm but ≤ 26.5 mm, and > 26.5 mm. We also classified patients into stages as a function of final outcome, defining patients with non-detached retina as stage 0, a non-detached retina treated with silicone oil as stage 1, a detached retina treated with silicone oil as stage 2 and phthisis bulbi as stage 3.

The study was approved by the ethics committee of Donostia University hospital, following the principles of the Declaration of Helsinki.

### Statistical analysis

The first step was to perform a descriptive analysis of the variables. Then, univariate analysis was performed to assess differences between the groups. For this purpose, Fisher’s exact test was used for dichotomous categorical variables when the expected values were less than or equal to 5, and otherwise, the chi-squared test. For continuous variables, means were compared using analysis of analysis for comparisons between three or more groups, and Student’s t-test for two-group comparisons when data were normally distributed. On the other hand, nonparametric variables were assessed using Kruskal–Wallis or Wilcoxon-Mann–Whitney tests for comparisons between three or more groups or two groups, respectively.

Finally, the normality of the data was tested using nonparametric methods such as the Kolmogorov–Smirnov test. The times between surgical procedures and the effects of various covariates were assessed by constructing Kaplan–Meier curves and Cox models. Further, logistic models were built to assess the likelihood of recurrence and analyze associated factors. The statistical analysis was carried out in various steps using R statistical software (version 4.1.2) and considering a 95% confidence interval.

## Results

We identified a total of 821 patients who underwent surgery for retinal detachment at Donostia University Hospital between 2012 and 2020. Forty per cent of these patients had psRD (n = 330), the others having phakic retinal detachment (pRD, n = 491). The characteristics of the population are summarized in Tables 1 and 2.

**Table 1:** Characteristics of patients with retinal detachment categorized by lens status (phakic or pseudophakic)

Variable	Values	Phakic	Pseudophakic	Total	p.value
Total	N	491 (59.81%)	330 (40.19%)	821 (100%)	
Gender	Female	176 (35.85%)	99 (30.00%)	275 (33.5%)	
	Male	315 (64.15%)	231 (70.00%)	546 (66.5%)	0.076
Relapse	No	364 (74.13%)	235 (71.21%)	599 (72.96%)	
	Yes	123 (25.05%)	95 (28.79%)	218 (26.55%)	
	Missing data	4 ( 0.81%)	0 ( 0.00%)	4 (0.49%)	0.136
Outcomes	Retina attached under Silicone oil	9 ( 1.83%)	14 ( 4.24%)	23 (2.8%)	
	Retinal detachment	21 ( 4.28%)	33 (10.00%)	54 (6.58%)	
	Retina attached	453 (92.26%)	274 (83.03%)	727 (88.55%)	
	Perfluoroctane toxicity	1 ( 0.20%)	4 ( 1.21%)	5 (0.61%)	
	Missing data	7 ( 1.43%)	5 ( 1.52%)	12 (1.46%)	0.001
RD Location	Inferior	124 (25.25%)	105 (31.82%)	229 (27.89%)	
	Nasal	16 ( 3.26%)	10 ( 3.03%)	26 (3.17%)	
	Superior	256 (51.93%)	145 (43.94%)	401 (48.72%)	
	Temporal	41 ( 8.35%)	26 ( 7.88%)	67 (8.16%)	
	Total	54 (11.00%)	44 (13.33%)	98 (11.94%)	0.001
Break Location	Multiple	23 ( 4.68%)	15 ( 4.55%)	38 (4.63%)	
	Inferior	114 (23.22%)	83 (25.15%)	197 (24%)	
	Missed break	54 ( 10,99%)	49 (14.85%)	103 (12,52%)	
	Superior	300 (61.09%)	183 (55.45%)	483 (54,20%)	0.000
Macula status	Off	308 (62.73%)	228 (69.09%)	536 (65.29%)	
	On	183 (37.27%)	102 (30.91%)	285 (34.71%)	0.066
	Scleral buckle + PhacoVPP	1 ( 0.20%)	0 ( 0.00%)	1 (0.12%)	
Tecnique	Scleral Buckle and VPP	49 ( 9.98%)	39 (11.82%)	88 (10.72%)	
	Scleral surgery	9 ( 1.83%)	1 ( 0.30%)	10 (1.22%)	
	PhacoVPP	30 ( 6.11%)	4 ( 1.21%)	34 (4.14%)	
	VPP + GAS	402 (81.87%)	286 (86.67%)	688 (83.8%)	0.002

**Table 2:** Variables in patients with retinal detachment, grouped according to the state of the lens

Variable	Values	Phakic	Pseudophakic	Total	p.value
Total	N	491 (59.81%)	330 (40.19%)	821 (100%)	
Age	< 45	61 (12.42%)	5 ( 1.52%)	66 (8.04%)	
	45–54	98 (19.96%)	42 (12.73%)	140 (17.05%)	
	55–64	177 (36.05%)	75 (22.73%)	252 (30.69%)	
	65–74	117 (23.83%)	118 (35.76%)	235 (28.62%)	
	> = 75	36 ( 7.33%)	90 (27.27%)	126 (15.35%)	
	Missed date	2 ( 0.41%)	0 ( 0.00%)	2 (0.24%)	0.000
Eye	Left	201 (40.94%)	161 (48.79%)	362 (44.09%)	
	Right	288 (58.66%)	169 (51.21%)	457 (55.66%)	
	Missed date	2 ( 0.41%)	0 ( 0.00%)	2 (0.24%)	0.034
Gauge	20	14 ( 2.44%)	8 ( 2.42%)	22 (2.44%)	
	23	202 (41.14%)	128 (38.79%)	330 (40.19%)	
	25	263 (53.56%)	193 (58.48%)	456 (55.54%)	
	Missing date	12 ( 2.44%)	1 ( 0.30%)	13 (1.58%)	0.117
	Air	2 ( 0.41%)	0 ( 0.00%)	2 (0.24%)	
Tamponade	Silicone Oil	30 ( 6.11%)	40 (12.12%)	70 (8.53%)	
	C2F6	7 ( 1.43%)	3 ( 0.91%)	10 (1.22%)	
	C3F8	69 (14.05%)	66 (20.00%)	135 (16.44%)	
	Hea	3 ( 0.61%)	2 ( 0.61%)	5 (0.61%)	
	SF6	371 (75.56%)	217 (65.76%)	588 (71.62%)	
	Missing date	9 ( 1.83%)	2 ( 0.61%)	11 (1.34%)	0.004
Previos vitreoretinopathy proliferative	No	473 (96.33%)	304 (92.12%)	777 (94.64%)	
	Yes	18 ( 3.67%)	26 ( 7.88%)	44 (5.36%)	0.015
	22 < = LA < 23.5	109 (22.20%)	84 (25.45%)	193 (23.51%)	
Axial lenghts (mm)	23.5 < = LA < 26.5	237 (48.27%)	135 (40.91%)	372 (45.31%)	
	26.5 < = LA < 29	91 (18.53%)	37 (11.21%)	128 (15.59%)	
	LA < 22	3 ( 0.61%)	5 ( 1.52%)	8 (0.97%)	
	LA > = 29	22 ( 4.48%)	14 ( 4.24%)	36 (4.38%)	
	Missing data	29 ( 5.91%)	55 (16.67%)	84 (10.23%)	0.000

In both groups, there were more men than women, with a somewhat higher number in the psRD group (70% vs 64.15%; p = 0.07). Regarding sex differences in eye size, men had longer ALs, though differences did not reach significance (25.47 ± 2.11 mm vs 24.59 ± 2.20 in women, p = 0.1087). Right eyes were slightly more affected than left eyes in both groups (58.56% in pRD group and 51,25% in psRD group)( p = 0.034).

The mean ages in the pRD and psRD groups were 58.34 ± 12.73 and 66.87 ± 11.18 years, respectively (p = 0.0001). Classifying patients by age group, the majority of psRD patients were in their fifth or sixth decade of life while the majority of pRD patients were in their sixth or seventh decade (Table 2).

In both groups, most patients had moderate myopia, defined as an AL of 23.5 to 26.5 mm (48.27% in pRD patients vs 40.91% in psRD patients) (Table 2). Further, the most common location for RD was the superior in both groups (43.94% in psRD patients vs 51,93% in pRD patients). the percentages of patients with RD involving the inferior retina or all four quadrants (total RD) were higher in the psRD group (31.82% and 13.33% of patients having lower and total RD, respectively vs 25.25% and 11.0% in the pRD group respectively). The tear was most commonly located in superior (between 3 and 9 o’clock) in both groups, with a somewhat higher rate in phakic patients (61.09% vs 55.45% in the psRD group). The rate of inferior tears (between 4 and 8 o’clock) was similar in the two groups, though slightly higher in pseudophakic patients (25.15% vs 23.22%). No tears were found in 14.85% of phakic and 10.99% of pseudophakic patients (p = 0.000).

There was macular involvement before surgery in the majority of patients in both groups, with a slightly higher rate in the psRD group (69.09% vs 62.04% in the pRD group) but the difference did not reach significance (p = 0.067).

Patients with had significantly longer ALs (by as much as 1 mm, 25.52 ± 2.14 mm vs 24.93 ± 2.27 mm in the PRD group; p = 0.019). The percentage of patients with proliferative vitreoretinopathy before surgery was higher in the psRD group (7.88% vs 3.6% in the pRD group; p = 0.01). The rate of recurrence after the first surgery was somewhat, though not significantly, but is higher in the psRD group (28.79% vs 25% in the pRD group; p = 0.17).

There were clear differences in the final anatomic outcome of patients who underwent surgery for RD in our series, the rate of final retinal reattachment being higher in pRD patients (94.09%) than in psRD patients (87.27%; p = 0.001). Table 3 show the main characteristics of patients with failure of final anatomic reattachment of retinal detachment after RD surgery (eyes with residual silicone oil or progressing to phthisis bulbi after the surgical procedures). We should highlight that there was macular involvement at diagnosis in 98.07% of these patients with poor anatomic outcome. In the pseudophakic RD group, 37.5% of patients with poor anatomic outcome had a history of cataract surgery complications. In the pRD group, more than half of the patients with final anatomic failure had total RD at diagnosis, compared to 28.12% in the pseudophakic RD group.

**Table 3:** Characteristics of final anatomical failure after Retinal Detachment (RD) surgeries. Persistence of DR under silicone oil

Characteristics of retinal anatomic failure after RD surgeries	Pseudophakic retinal detachment ( n = 32)	Phakic retinal detachment (n = 21)	Total (N = 53)	p value
Female ( n (%))	12 (37,5)	9(42,85)	21	
Male ( n (%))	20 (62,5)	12(57,14)	32	**0.569**
Inferior RD( n (%))	17(53,12)	6(28,57)	23	
Superior RD( n (%))	6 (18,75)	4(19,04)	10	
Total RD	9 (28,12)	11 (52,38)	20	**0.093**
Missed break ( n (%))	12(37,5)	5(23,8)	17	**0.004**
Macula ON ( n (%))	1(3,12)	0 (0)	1	
Macula OFF( n (%))	31 (96,87)	21 ( 100)	52	**1.000**
History of complicated pseudophakia n (%))	12 (37,5)	–	–	

A significance p value (p<0.05) was found in "missed break" variable, being more frequent in pseudophakic patients

## Discussion

Rhegmatogenous RD is a condition caused by a full-thickness retinal hole or tear usually produced by vitreous traction secondary to PVD that leads to the separation of the neurosensory retina from the retinal pigment epithelium [8] The incidence of rhegmatogenous RD is around 0.08% in the general population and tenfold higher in patients with a history of cataract surgery.

Humans are the only species to have vitreous degradation over their lifetime. This degeneration leads to the development of PVD. With progressive collagen condensation in the vitreous body, there is an increase in the volume of liquid vitreous, a process known as vitreous syneresis. [10, 11] These are physiological changes associated with ageing of the vitreous body and start in the fourth decade of life, peaking between the sixth and seventh decade, coinciding with the highest peak of RD incidence in phakic patients, as observed in our study [2].

Vitreous liquefaction alone, however, does not lead to complete vitreous detachment, this also requiring a weakening of vitreous cortex-to-internal limiting membrane adhesion. This slow physiological process is interrupted during phacoemulsification, due to anatomic and biochemical changes secondary to cataract surgery, inducing vitreous detachment with stronger traction, since there is a lack of prior weakening of the attachment of the vitreous cortex to the periphery of the retina, making retinal tears more likely [12–15]. For this reason, having complete PVD prior to cataract surgery is considered protective against pseudophakic RD.

The different pathogenic mechanisms in pseudophakic patients mean that the visual prognosis varies as a function of the status of the crystalline lens. Our study also found poorer outcomes in patients with a history of cataract surgery, as in the scientific literature: it seems likely that differences in the pathogenic mechanism result in a type of RD that is characterized by a poorer course. [16, 17].

Regarding sex, both of our study groups were dominated by men, consistent with the scientific literature, this being especially marked in the pseudophakic group, as in the series analyzed by Lumi et al. [18]. The reason for a higher incidence in men is still unclear, although it has been suggested that it may be related to a greater risk of trauma in men and potential anatomic differences that make them more susceptible. In our series, we did not find sex differences in AL that might explain this predominance in men found in both study groups.

In our study, we found that patients under 60 years of age were more than one third of the sample in pseudophakic patients, probably because they lack the protective effect of PVD at the time of phacoemulsifcation; that is, the younger patients are when undergoing cataract surgery, the less likely they are to have full-thickness PVD. Among phakic patients, there is a higher prevalence of RD at the peak of the PVD incidence, between 60 and 70 years old.

Physiological PVD has been found to start in the temporal visual field [19], and peripheral retinal degeneration usually appears in the superior temporal area [20]. Given this, we would expect RD to mostly occur in the superior temporal quadrant, as found in our sample in both study groups. Rates of inferior and four-quadrant RD were higher in the pseudophakic patients than in phakic patients, as found in the scientific literature. Nonetheless, analyzing inferior tears, defined as those between 4 and 8 o’clock [21], we found similar rates in the two groups. In our sample, pseudophakic patients had more tears that were not found at the time of the surgical procedure, occult tears also being associated with failure in surgery. Patients with anatomic failure after surgery had higher rates of tears not found intraoperatively and total and lower RD, as can be seen in Table 3.

Although the difference was not statistically significant, macular involvement at the time of surgery was also more common among pseudophakic RD patients. Anomalous PVD observed in these patients after phacoemulsification surgery would result in stronger traction at the periphery, in turn explaining the greater extent of RD and greater risk of macular involvement [22]. A surgical complication at the time of surgery is a known risk factor for RD [23], and notably, in our sample, more than a third of patients with a poor final anatomic outcome had a history of posterior capsular rupture during phacoemulsification.

The aforementioned features (inferior RD/tears, total RD, and macular involvement at the time of surgery) increase the risk of recurrence after the first surgery. In our sample, after analyzing patients with final anatomic failure, this structural outcome was more common in patients with a detached macula at the time of diagnosis and total RD. Inferior tears have also been associated with a poorer final anatomic response. The explanations suggested for these findings include a weaker effect of the tamponade and also vitreous shaving being more difficult in the lower half of the eye, especially in phakic patients.

The rate of primary reattachment after the primary surgery ranges from 71 to 94% depending on the series analyzed [23]. In our sample, the risk of recurrence was greater in pseudophakic patients (28.79%), although the difference did not reach significance.

Regarding the limitations of this study, we should indicate that it was retrospective, with the higher rate of missing data this implies, given the lack of an established protocol for the entry of data in patient health records covering all the parameters we would like to have analyzed in this subsequent analysis. In addition, this was a single-center study in a tertiary hospital in the Donostialdea Health Region, and hence, the number of patients that could potentially be recruited was limited to cases among the catchment population of this region. Other limitation of the study, is the lack of information about the presence or absence of PVD prior to phacoemulsification in those patients from the psRD group, although in both groups of regmatogenous RD it´s highly probable to be secondary to an acute PVD some days or weeks before developing RD.

To conclude, in our study, pseudophakic patients had poorer outcomes than phakic patients and greater likelihood of final anatomic failure. It is likely that the mechanism of pathogenesis secondary to anomalous PVD seen in patients after cataract surgery is the reason pseudophakic patients have RD characterized by a poorer course. Further comparison studies are required to confirm these findings.

## Data Availability

The datasets during and analysed during the current study available from the corresponding author on reasonable request.
